# Ethnic and adoption attitudes among Guatemalan University students

**DOI:** 10.1186/s40064-015-1578-2

**Published:** 2015-12-18

**Authors:** Judith L. Gibbons, Ana Gabriela González-Oliva, Kostas Mylonas

**Affiliations:** Department of Psychology, Saint Louis University, Morrissey Hall 2826, 3700 Lindell Blvd., St. Louis, MO 63108 USA; Department of Psychology, Universidad del Valle de Guatemala, 18 ave. 11-95 zona 15 Vista Hermosa III Oficina G203, Guatemala, Guatemala; Psychology Faculty, National and Kapodistrian University of Athens, Panepistimiopolis, ILISIA, 15784 Athens, Greece; Antigua, Guatemala, Central America

**Keywords:** Adoption, Guatemala, Ethic attitudes, Interethnic adoption, Subsidiarity clause of the Hague Convention

## Abstract

Intercountry adoptions from Guatemala were highly controversial, because of the large numbers of children being adopted to the USA, along with evidence of corruption and child theft. Since the implementation of the Hague Convention on Intercountry Adoption in 2008, Guatemala’s central authority for adoption has prioritized domestic placements for children over intercountry adoption. A possible attitudinal barrier to domestic adoption in Guatemala—negative attitudes and prejudice against Indigenous people—was investigated through questionnaires measuring attitudes toward adoption and attitudes toward and social distance from the two major ethnic groups (Ladino and Indigenous). Guatemalan university students (*N* = 177, 61 % men) were recruited from basic required courses at a private university. Results showed that attitudes toward adoption in general were more favorable than toward interethnic adoption, with the most negative attitudes toward adoption of Ladino children by Indigenous parents. Multiple regression and analysis of covariance models revealed that female gender, experience with adoption and more positive attitudes about Indigenous persons were associated with more positive attitudes toward adoption. The findings imply that negative attitudes toward Indigenous persons are associated with negative attitudes toward adoption, and serve as barriers to promoting domestic adoption in Guatemala.

## Background

Guatemala was at the center of the controversy surrounding intercountry adoption (ICA). In 2007 at the peak of Guatemala’s role as a sending country, one percent of the babies born in Guatemala were being relinquished for adoption by foreigners, mostly from the United States (Selman 2012). Reports of child theft, sale, and trafficking roused international consternation (Bunkers and Groza 2012; Comisión Internacional Contra la Impunidad en Guatemala, CICIG 2010; Goicoechea and Degeling 2007; Rotabi 2012; Rotabi et al. 2008) and subsequently, one case of child abduction for ICA was proven with DNA testing (“Bebé Robada” 2008; Bunkers et al. 2009). In 2008, Guatemala implemented the Hague Convention on the Protection of Children and Co-operation in Respect of Intercountry Adoption, known as the Hague Convention (Hague Conference on Private International Law 1993). This international agreement is designed to protect children’s well-being and to prevent abuses such as those reported in Guatemala.

In 2008 Guatemala’s newly formed central agency, the Consejo Nacional de Adopciones (CNA) processed only 16 domestic (national) adoptions, and 27 new foreign adoptions, to countries other than the USA (Equipo de investigación 2008). To date, the agency continues to process, after review, adoptions that were in progress before the implementation of the Hague Convention. Since 2008 the CNA has strived to encourage adoption within Guatemala and has completed the processing of 64–184 domestic adoptions per year from 2008 through 2013 (Contraloría General de Cuentas 2014; Groza and Bunkers 2014).

Based on the guidelines of the Convention on the Rights of the Child (Office of the United Nations High Commissioner for Human Rights 1989), and the conviction that every child deserves a family, the Hague Convention requires a stepwise process known as the subsidiarity principle. Local options, including placement within the child’s extended family, as well as domestic adoption, must be exhausted before turning to ICA placement. The Guatemalan law reads: “International adoption can only proceed after appropriate consideration of possibilities for national (domestic) adoption” (Congreso de la República de Guatemala 2007; Equipo de Investigación 2008). Because formal adoption among Guatemalans has been extremely rare in the past (Bunkers et al. 2009) additional ways of promoting and facilitating domestic adoption must be pursued. A step towards this goal is identifying the barriers, both procedural and attitudinal, to domestic adoption of children. The CNA might then address those barriers to find adoptive placements for the over 4000 children currently living in institutions in Guatemala (Groza and Bunkers 2014).

Most Guatemalans agree that domestic adoptions are preferable to intercountry adoptions. In a poll of 842 adults by the Guatemalan daily newspaper *Prensa Libr*e, the majority (51.2 %) responded that a child would be better off adopted by a Guatemalan family; fewer (40.5 %) responded that the child would be better off in a foreign country (Seijo 2008). When asked whether adopted children would be *happier* with a Guatemalan family or with a foreign family, 55 % replied that they would be happier with a Guatemalan family and 37.1 % replied that they would be happier with a foreign family (Seijo 2008).

Some of the barriers to domestic adoption in Guatemala are societal and structural—widespread poverty, a high birth rate, and lack of knowledge or publicity about the legal requirements and procedures for adopting (Bunkers et al. 2009; Gibbons et al. 2009; Wilson and Gibbons 2005). The lack of information about the adoption process is currently being addressed by the CNA; this entity has initiated efforts to promote domestic adoption and to educate the public through a website, facebook page, and free seminars and workshops aimed at prospective adoptive parents.

But attitudinal barriers may also play a role. For example, in a number of studies women have been shown to hold more positive attitudes toward adoption than do men (e.g., Evan B. Donaldson 2002). Both in Guatemala and the United States the gender difference in attitudes was mediated by machismo, the endorsement of an extreme masculinity along with sexist beliefs about women (Gibbons et al. 2006a, b). Persons who held more egalitarian gender role attitudes and endorsed machismo less were more positive about adoption.

A second attitudinal barrier may be ethnic prejudice. There are two major ethnic groups in Guatemala—Ladinos, who are persons of mixed European and Indigenous heritage and Indigenous, most of whom are of Mayan descent and speak one of the 22 Mayan languages. Discrimination against Indigenous people, although prohibited by law, is evident in economic, educational, and health care domains (Programa de las Naciones Unidas para el Desarrollo Humano, PNUD 2005). For example, 80 % of Indigenous people in Guatemala live in poverty or extreme poverty, compared to 45 % of non-Indigenous people. In addition, Ladinos and Indigenous tend to hold mutually negative attitudes, with a majority of each group claiming that people of the other ethnicity are less agreeable, less intelligent, and less honest than members of their own group (PNUD 2005).

In several interview studies respondents identified racism in Guatemala as a potential attitudinal barrier to adoption. “Here [in Guatemala] many people are prejudiced,” was a comment by a Guatemalan interviewee (Gibbons et al. 2009 p. 69). Similar views were expressed in a second study, in which a respondent said, “the mentality is a race issue…they [Guatemalans] won’t adopt because of race or looks of a child” (Wilson and Gibbons 2005 p. 749).

Nevertheless, in the poll of 842 Guatemalan adults reported in the newspaper *Prensa Libre* most denied that they would object to adopting an Indigenous child (Seijo 2008). In answer to the question, “would it worry you a great deal, somewhat a little, or almost not at all, if [your adopted child] were Indigenous?” only 5 % reported “somewhat or a great deal” and 92.6 % said “almost not at all”.

Despite the denial by the majority of Guatemalan adults of the importance of ethnicity in influencing their willingness to adopt, adoption attitudes are known to be embedded in people’s social attitudes, cultural assumptions, and beliefs (Bausch 2006; Evan B. Donaldson Institute 2002; Hollingsworth 2000). Because of the documented prejudice and discrimination in Guatemala against Indigenous persons (e.g. Hale 2006), we set out to investigate whether attitudes about adoption differed for inter-ethnic adoption and adoption in general, and also whether attitudes toward the two ethnic groups in Guatemala were related to attitudes about adoption.

## Method

### Participants

The participants were 177 students (108 men, 68 women, 1 missing gender) at a private university located in Guatemala City. They were recruited through general required classes, usually taken in the first year of study. Ethnic identification was measured on a 14 mm line labeled “pure Indigenous” at one end and “pure Ladino” at the other; participants who could or would not place themselves on the line could write their ethnicity in a blank space. Six participants did not locate themselves on the line, but wrote Chinese (1) or from the USA (2) or did not answer (3). Characteristics of the participants are presented in Table 1.

**Table 1:** Characteristics of the participants

Characteristic	Value
Age (years)	*M* = 18.4, *SD* = 1.07, range = 18 through 28
Gender (%)	
Male	61.4
Female	38.6
Year in school (%)	
First year	92.6
Second year	2.8
Third year or more	4.6
Ethnicity (from 0, Indigenous pole to 14, ladino pole)	*M* = 11.84, *SD* = 2.00
Religion (%)	
Roman Catholic	79.7
Other Christian	9.9
Other	10.5
Marital status (%)	
Single	99.4
Divorced	0.6
Father’s occupation (%)	
Professional	89.5
Non-professional	10.5
Mother’s occupation (%)	
Professional	63.9
Non-professional	15.7
Homemaker	20.5
Experience with adoption (%)	54.6
Women	64.2
Men	48.1

### Measures and procedure

The 10-item Multigroup Ethnic Identity Measure (MEIM-S, Yancey et al. 2001) was used as a measure of ethnic identity. This measure has shown good reliability among diverse youth in the USA. A sample item is, “I have a lot of pride in my ethnic group and its accomplishments” (Phinney 1992). Cronbach’s alpha coefficient ranged from 0.78 to 0.83 among the four ethnic groups tested in the USA. In the present study, Cronbach’s alpha reached 0.78.

The Social Distance Scale was a 9-item version based on the original Social Distance Scale (Bogardus 1932) as modified by Byrnes and Kiger (1988). Variations of this scale have enjoyed wide use in countries as divergent as Pakistan (Zaidi 1967) and Fiji (Thomas 1974). Participants responded from 1 labeled *very uncomfortable* to 7 *very comfortable* the degree to which they felt comfortable having a Ladino (Indigenous) person as a dance partner, the governor of their state, etc. The Byrnes and Kiger (1988) version was modified by replacing the United States with Guatemala in the item, “president of….” An additional item was added for the present study about “my adopted child.” The mean response was used for data analysis, with higher scores representing greater comfort (less social distance). In the present study, this version of the Social Distance Scale showed good reliability; Cronbach’s alpha was 0.89 for the Ladino version and 0.83 for the Indigenous version.

The AIG (Attitudes toward Indigenous of Guatemala) is a 23-item scale (Ashdown et al. 2011; Gibbons and Ashdown 2010) that was developed to measure attitudes toward Indigenous persons of Guatemala. Responses are made on a 4-point scale from *strongly agree* to *strongly disagree* to items, such as “The majority of the Indigenous population is hardworking.” Higher scores represent more positive attitudes. Cronbach’s alpha in the development of the scale was 0.84 (Gibbons and Ashdown 2010) and in the present study was 0.83.

The ALG (Attitudes toward Ladinos of Guatemala) is a 14-item scale designed to measure attitudes toward the Ladino ethnic group in Guatemala. Items such as “Ladinos deserve a good economic situation because of their effort” are rated on a 4-point scale from *strongly agree* to *strongly disagree*. Higher scores represent more positive attitudes. Cronbach’s alpha in the development of the scale was 0.79 (Gibbons and Ashdown 2010) and in the present study it was 0.68.

The Adoption Beliefs Scale (ABS, Gibbons et al. 2006b) is an 11-item scale designed to measure adoption attitudes. Its utility was first demonstrated in Guatemala, although it has shown good reliability and validity in the USA as well. Among a sample of Guatemalan university students the alpha was 0.70 (Gibbons et al. 2006b), and among a sample of USA university students, the alpha was 0.79 (Gibbons et al. 2006a). In the present study one item, “Both the birthparents and adoptive parents are real parents,” failed to correlate with the total score, and it was replaced by the item, “Adoption serves a useful purpose in our society” from the Evan B. Donaldson (1997) Benchmark Survey. This modified ABS had an alpha of 0.68.

In addition, items adapted from the Evan B. Donaldson (1997) survey were used to assess general attitudes about adoption and specifically about international adoption, inter-ethnic adoption by Ladinos, and inter-ethnic adoption by Indigenous. The basic item was “In general do you have a very favorable opinion of adoption, a somewhat favorable opinion, a somewhat unfavorable opinion, or a very unfavorable opinion of adoption?” Responses were “*very favorable*,” “*somewhat favorable*,” “*somewhat unfavorable*”, and “*very unfavorable*.” In addition, similar questions that specified the type of adoption–adoption of Guatemalan children by foreigners (intercountry adoption), adoption of Indigenous children by Ladinos, and adoption of Ladino children by Indigenous, were specified. Participants were also queried about their experience with adoption, using the Evan B. Donaldson question, “Has anyone in your family, or among your close friends, ever been adopted OR adopted a child OR placed a child for adoption?”.

Some of the instruments, including the ABS, the AIG, and the ALG, had been developed in Spanish. The remainder of the instruments and questions were subjected to a rigorous translation procedure. They were first translated from English to Spanish by a native Spanish speaker bilingual in English. Those translations were back-translated, checked and revised by a native English speaker bilingual in Spanish. Those two authors, both bilingual, reconciled discrepancies.

Participants were invited to participate through use of a recruitment statement approved by the Institutional Review Board of the first author’s university. Participation was voluntary and anonymous. Questionnaires were distributed in classrooms, and at the discretion of the instructor, students completed them in class or took them home and returned them during the following class period.

## Results

### Experience with adoption

Of the 174 participants who answered the query about experience with adoption, 95 (54.6 %) reported that at least one family member or close friend was a member of the adoption triad (adoptee, adoptive parent, or birth parent). Women (64.2 %) reported more experience with adoption than did men, (48.1 %), χ^2^ (1, *N* = 173) = 4.27, *p* < 0.05. Experience with adoption was related to more favorable attitudes toward adoption, as measured using the modified ABS, *t* (172) = 2.30, *p* < 0.05, η^2^ = 0.03, and also using the single item as a measure of favorability, *t* (169) = 3.02, *p* < 0.01, η^2^ = 0.05. The items about international adoption, *t* (172) = 2.40, *p* < 0.05, η^2^ = 0.03, inter-ethnic adoption by Ladinos, *t* (171) = 2.58, *p* < 0.05, η^2^ = 0.04, and inter-ethnic adoption by Indigenous, *t* (171) = 2.85, *p* < 0.05, η^2^ = 0.03, also showed an effect of experience, with participants who had experience with adoption expressing more favorable views. In summary, “experience with adoption” accounted for between 3 and 5 % of the variance in attitudes toward adoption.

### Gender differences

There were significant gender differences with respect to three measures. Women showed more positive attitudes toward indigenous on the AIG. They also reported more favorable attitudes toward adoption on both the modified ABS and on the single question about adoption in general. See Table 2 for the means and standard deviations of the study variables by gender.

**Table 2:** Mean scores (and standard deviations) on predictor and dependent variables by gender

Variable	Possible range of scores	Women	Men	*t*	*p*	95 % CI_Δ_
Ethnicity	0–14	12.04 (2.04)	11.70 (1.99)	1.04	NS	[−0.96, 0.30]
MEIM-S	1–4	2.00 (0.40)	2.06 (0.46)	0.82	NS	[−0.08, 0.19]
Social distance from Ladinos	1–7	6.04 (0.95)	5.87 (0.97)	1.12	NS	[−0.46, 0.12]
Social distance from Indigenous	1–7	4.26 (1.50)	4.15 (1.26)	0.51	NS	[−0.52, 0.31]
ALG	1–4	2.91 (0.35)	2.90 (0.35)	0.19	NS	[−0.12, 0.10]
AIG	1–4	3.06 (0.34)	2.92 (0.35)	2.58	<0.05	[−0.24, −0.03]
ABS (modified)	1–4	3.12 (0.39)	2.97 (0.36)	3.72	<0.001	[−0.33, −0.10]
Adoption in general	1–4	3.73 (0.45)	3.54 (0.56)	2.31	<0.05	[0.03, 0.33]
International adoption	1–4	3.61 (0.60)	3.48 (0.75)	1.21	NS	[−0.33, −0.03]
Interethnic adoption by Ladinos	1–4	3.52 (0.59)	3.33 (0.66)	1.91	NS	[−0.38, 0.01]
Interethnic adoption by Indigenous	1–4	3.22 (0.79)	2.99 (0.74)	1.96	NS	[−0.47, 0.00]

*MEIM-S* multigroup ethnic identity measure, short-form, *ALG* attitudes toward Ladinos of Guatemala, *AIG* attitudes toward Indigenous of Guatemala, *Social distance* higher numbers indicate less social distance, *NS* not significant, *95* *% CI*_*Δ*_ 95 % confidence interval of the difference in means

### Favorability toward adoption

In response to the favorability of the different kinds of adoption, there were also significant differences. A repeated measures ANOVA demonstrated a significant main effect for type of adoption, *F* (3, 167) = 32.02, *p* < 0.001, partial η^2^ = 0.16, with the most favorable attitudes expressed toward adoption in general (*M* = 3.61 ± 0.52), followed by international adoption (*M* = 3.53 ± 0.69), adoption of Indigenous by Ladinos (*M* = 3.40 ± 0.64), and adoption of Ladinos by Indigenous (*M* = 3.08 ± 0.77); t-tests were conducted as post-hocs using the Bonferroni procedure to correct for multiple tests. Interethnic adoption of Indigenous by Ladinos was viewed significantly less favorably than adoption in general, *t* (169) = 3.86, *p* < 0.001, 95 % CI_Δ_ = [0.09, 0.29], and adoption of Ladinos by Indigenous was viewed less favorably than all other kinds of adoption, including adoption in general, *t* (169) = 8.37, *p* < 0.001, CI_Δ_ [0.40, 0.65], intercountry adoption, *t* (172) = 6.72, *p* < 0.001, 95 % CI_Δ_ [0.31, 0.58], and adoption of Indigenous by Ladinos, *t* (172) = 5.96, *p* < 0.001, 95 % CI_Δ_ [0.22, 0.43].

### Predictors of attitudes toward adoption

A multiple regression analysis was used to determine the correlates (predictors) of the criterion variable, the modified ABS. The predictor variables were entered simultaneously in the model as independent predictors. The predictors were ethnic identification, ethnic identity as measured by the MEIM-S, the product of those two variables expressing their interaction, the attitudinal variables AIG and ALG, and the two social distance scales. The model was significant, *R* = 0.69, *R*^*2*^ = 0.14, Adjusted *R*^*2*^ = 0.10, *F* (7, 151) = 3.38, *p* < 0.01. Attitudes toward indigenous people contributed significantly to the model, and predicted more positive attitudes toward adoption. Table 3 shows the zero order correlations among the predictor and criterion variables. Table 4 presents the results of the multiple regression analysis (intercept = 2.92).

**Table 3:** Correlations among the variables (N = 165–177)

Variables	1	2	3	4	5	6	7	8	9	10	11
1. Adoption beliefs scale	–										
2. Adoption in general	0.44***	–									
3. International adoption	0.38***	0.40**	–								
4. Interethnic by Ladinos	0.38**	0.34**	0.42**	–							
5. Interethnic by Indigenous	0.25**	0.25**	0.29**	0.49**	–						
6. Ethnicity	0.12	0.08	0.14	0.00	0.22**	–					
7. MEIM-S	−0.09	0.07	−0.07	0.06	−0.05	−0.07	–				
8. Social distance from Ladinos	0.21**	0.15	0.09	0.17*	0.05	0.31**	−0.03	–			
9. Social distance from Indigenous	0.27**	0.14	0.13	0.26**	0.44**	−0.05	−0.08	0.26**	–		
10. ALG	0.08	0.16*	0.08	0.00	−0.02	0.27**	−0.11	0.28**	−0.02	–	
11. AIG	0.30**	0.03	0.02	0.23**	0.23**	−0.21**	−0.15*	0.01	0.51**	−0.13	–

*Ethnicity* higher indicates closer to Ladino pole, *Social distance* higher numbers indicate less distance

* *p* < 0.05, ** *p* < 0.01, *** *p* < 0.001

**Table 4:** Multiple regression analysis: modified ABS regressed on the predictor variables

Predictor variables	Modified ABS as criterion variable (N = 158)		
	*B*	*SE*	β
Ethnicity	−0.10	0.09	−0.50
Ethnic Identity (MEIM-S)	−0.62	0.53	−0.73
Ethnicity * MEIM-S	0.05	0.04	0.88
Social distance from Ladinos	0.03	0.03	0.07
Social distance from Indigenous	0.04	0.03	0.14
ALG	0.11	0.09	0.11
AIG	0.25	0.10	0.24*
R^2^			0.14
R^2^ adjusted			0.10

*Ethnicity* measured on a continuous scale from 0 to 14 with higher numbers representing more Ladino identity, *MEIM-S* multigroup ethnic identity measure, short-form, *ALG* attitudes toward Ladinos of Guatemala, *AIG* attitudes toward Indigenous of Guatemala, *Social distance* higher numbers indicate less social distance

* *p* < 0.05, ** *p* < 0.01, *** *p* < 0.001

Because of their demonstrated relation to adoption attitudes, gender and adoption experience were analyzed by means of an analysis of covariance (ANCOVA). The independent variables were gender (1 = male, 2 = female) and adoption experience (1 = yes, 2 = no) the covariate was attitudes toward indigenous (AIG, the significant predictor in the regression model), and the dependent variable was attitudes toward adoption as measured by the modified ABS. As expected, the AIG covariate was significant, *F* (1, 166) = 12.02, *p* < 0.001, partial η^2^ = 0.068. After controlling for attitudes toward indigenous people, gender also had a significant effect, with women reporting more positive attitudes than did men, *F* (1, 166) = 11.13, *p* < 0.001, partial η^2^ = 0.063. Adoption experience was significantly related to adoption attitudes with those having experience expressing more positive attitudes, *F* (1, 166) = 5.45, *p* < 0.05, partial η^2^ = 0.032. There was no significant interaction between the independent variables.

## Discussion

Overall, the participants in the present study expressed favorable attitudes toward adoption in general and toward inter-ethnic adoption. Adoption in general was most favorably viewed, followed by international adoption, with adoption of Ladinos by Indigenous viewed least favorably. But even in this last category, there was broad approval, with 76 % of the respondents expressing approval rather than disapproval. This overwhelming support for inter-ethnic or interracial adoption among university students may be widespread (at least when based on self-report) as evidenced in studies from the USA (Whatley et al. 2003) and South Africa (Moos and Mwaba 2007). In a study from the USA, university students reported low levels of modern racism; however, when they viewed a photograph of a transracial adoptive family, they endorsed more negative views of adoption than when they viewed a same-race adoptive family (Katz and Doyle 2013).

In the present study 55 % of the participants reported some experience with adoption, either among family members or close friends. Although this number is lower than the two-thirds typically reported among USA respondents (Evan B. Donaldson 2002), it still represents a majority of this sample, and suggests that many Guatemalan university students have some familiarity with adoptive relationships. Moreover, as demonstrated in other studies, those with adoption experience held more positive attitudes about adoption (Bausch 2006).

The results of the present study demonstrated that attitudes about adoption, including inter-ethnic adoption, were linked to attitudes toward Indigenous persons. Specifically, participants who held more positive attitudes toward Indigenous people reported more favorable attitudes toward adoption. Those results imply that ethnic attitudes are linked to attitudes about adoption within Guatemala, as they were among USA college students (Katz and Doyle 2013).

Neither attitudes toward Ladino persons, nor social distance from either of the ethnic groups was associated with attitudes toward adoption. Adoption attitudes were also not associated with participants’ own ethnic identification, although this sample was highly identified with Ladino ethnicity. As in most other studies of gender and adoption attitudes (Evan B. Donaldson Institute 2002; Gibbons et al. 2006a, b), women held more favorable attitudes toward adoption than did men, and female gender was associated with more positive attitudes toward adoption as the analysis of covariance revealed.

There are several limitations to the present study, including the use of university students as participants. In Guatemala university students represent a highly privileged sample, as only 4 % of the population has the opportunity to receive a university education (PNUD 2005). Nevertheless, the educational elite of Guatemala represents the population that is mostly likely to have the resources to adopt children, and thus their attitudes have implications for adoption policy.

A second limitation stems from the correlational nature of the data. Although there was a clear demonstration of a relationship between attitudes about adoption and attitudes toward Indigenous persons, this does not imply that negative attitudes toward Indigenous cause individuals to be less favorable toward adoption. Most likely individuals’ cognitive structures of attitudes and beliefs are linked to each other through relations with their more fundamental assumptions about life (Bond et al. 2004).

The third issue that needs attention is the apparent discrepancy between the present results that point to ethnic prejudice as a possible barrier to domestic adoption within Guatemala, and the national survey in which almost all respondents denied that they would be concerned about adopting a child of Indigenous origin (Seijo 2008). This apparent discrepancy is diminished by noting that, first, the majority of the respondents in the present study expressed approval of inter-ethnic adoption, and second, that there may be strong pulls for social desirability present in a telephone or face-to-face survey that are less pronounced in an anonymous questionnaire. The fact that so many Guatemalans denied ethnic prejudice in the *Prensa Libre* survey is a positive sign, if only to demonstrate that ethnic prejudice is not socially acceptable.

The present study carries with it implications for child welfare practice in Guatemala. As the CNA continues to promote domestic adoptions as part of a child welfare initiative, it would behoove the agency to coordinate their efforts with other systematic efforts to reduce racism in Guatemala. Examples of potential collaborators include the presidential commission,—*Comisión Presidencial contra la Discriminación y el Racismo* [Presidential Commission against Discrimination and Racism] (CODISRA; Presidencia de la República 2002) that was charged with investigating and developing plans to eliminate discrimination and racism in Guatemala. Another potential collaborator is the prominent non-profit foundation *El Centro de Investigaciones Regionales de Mesoamérica* (CIRMA) that sponsored an exhibit and a book series titled *Por qué estamos como estamos* [Why we are like we are] in which ethnicity in Guatemala was highlighted. Finally, educational programs aimed at training prospective adoptive parents in Guatemala might include segments on promoting cultural diversity as well.

## Conclusion

In conclusion, the present study has identified a possible attitudinal barrier to promoting domestic adoption in Guatemala—ethnic prejudice against Indigenous people. Efforts to reduce the structural barriers that impede domestic adoption might well be augmented by addressing attitudinal barriers, such as machismo and ethnic prejudice, in order to improve the lives of Guatemalan children who are in need of a family. As other countries implement the subsidiarity clause of the Hague Convention, their central adoption authorities would also benefit from examining and countering local prejudices that could stand in the way of finding homes for children in need of families.
